# WHO’**s** pandemic response recommendations after COVID-19: lessons learned or learnings lost?

**DOI:** 10.3389/fpubh.2025.1664330

**Published:** 2025-10-28

**Authors:** Jean Merlin von Agris, David Bell, Blagovesta Tacheva, Garrett Wallace Brown

**Affiliations:** ^1^School of Politics and International Studies, University of Leeds, Leeds, United Kingdom; ^2^Independent Consultant, Lake Jackson, TX, United States

**Keywords:** non-pharmaceutical interventions, public health and social measures, pandemic preparedness, pandemic response, World Health Organization, quarantine, masking, contact tracing

## Abstract

**Objectives:**

This article examines how the World Health Organization’s (WHO) recommendations and guidelines on public health and social measures (PHSM) have changed since COVID-19. Doing so allows insights on what lessons WHO has learned from the COVID-19 response.

**Methods:**

The article analyses six recent WHO publications detailing recommendations on PHSM and compares them against three pre-COVID-19 WHO documents. The analysis also assesses the evidence-base used for these recommendations to better understand WHO’s substantive basis and rationale for the PHSM changes.

**Results:**

The analysis reveals substantial changes in WHO recommendations, often without systematic evidence assessment. Several population-wide interventions including quarantine, travel measures, and universal masking have become normalized in post-COVID documents, despite being previously discouraged. When evidence is cited, it often pertains to narrowly defined short-term outcomes, with limited consideration of broader societal impacts. Adverse effects of PHSM are recognized, but mitigation takes priority over avoiding harms.

**Conclusion:**

Systematic evaluation of the evidence on PHSM during the COVID-19 pandemic, including their effectiveness and collateral effects, is imperative before revising changes in recommendations for future pandemics.

## Introduction

In the wake of COVID-19, pandemic prevention, preparedness, and response (PPPR) has gained center-stage in public health policy development. While negotiations around the World Health Organization’s (WHO) Pandemic Agreement (1) mainly revolved around improving access to medical countermeasures and several new institutions like the Pandemic Fund (2) and the Pandemic Hub (3) focus on pathogen surveillance, non-pharmaceutical interventions (NPI), or public health and social measures (PHSM), are likewise being reevaluated.

The terms NPI and PHSM are often used interchangeably. According to WHO, “PHSM refer to non-pharmaceutical interventions implemented by individuals, communities, and governments to reduce the spread of infectious diseases with epidemic or pandemic potential by reducing transmission of the pathogen” (4). WHO sees PHSM as buying time to develop specific responses to a pathogen, “decreasing the burden on health systems so that essential health services can continue and effective vaccines and therapeutics can be developed and deployed” (5).

As a vaccine, once developed, may not provide transmission-blocking or lasting immunity (6, 7), the justification for PHSM may not change after deployment of vaccination. In 2021, mask mandates, travel restrictions, and other PHSM widely remained in place after mass vaccination for COVID-19 (8, 9). These PHSM were often imposed without public health precedent and driven by imitation rather than evidence (10–12), contrary to earlier WHO recommendations for pandemic influenza, a virus with similar transmission characteristics and overall severity (13, 14).

As the principal norm-setting institution of global health, WHO issues non-binding recommendations to Member States (15). For development of its official guidelines, WHO ostensibly follows a rigorous internal quality assurance process including the Grading of Recommendations, Assessment, Development and Evaluation (GRADE) approach, rating the quality of evidence used (high, moderate, low, or very low). Yet, although WHO defines guidelines “broadly as any information product developed by WHO that contains recommendations for clinical practice or public health policy” (16), many WHO publications do not fully adhere to these procedures, including recommendations issued by emergency committees during a public health emergency of international concern (PHEIC). For example, WHO initially advised against “any travel or trade restrictions” during the COVID-19 PHEIC, consistent with their prior recommendations, but dropped this advice after most countries ignored it (17). Similarly, WHO recommended masking in the general population only after countries across the globe had introduced mask mandates (18, 19).

Following a resolution at the 2021 World Health Assembly (20), WHO launched an initiative to measure the effectiveness and impact of PHSM (21). The 2025 World Health Assembly recently reinforced the process (22). As part of this remit, WHO is re-examining its recommendations on PHSM to reflect the lessons from COVID-19. The WHO secretariat has begun meeting with national stakeholders in 2023 to deliberate on a conceptual framework for PHSM research and monitoring (23–25), which aims to support a research agenda to be completed by 2030 (26, 27). In August 2024, WHO designated the Center for Epidemic Interventions Research at the Norwegian Institute of Public Health as a WHO Collaborating Center for research on PHSM effectiveness (28).

WHO also set up its “PHSM Knowledge Hub” website in April 2024, featuring four interconnected tools for decision makers (27). First, a continuously updated bibliographic library of PHSM research. Second, the “PHSM Research Atlas” encompassing the PHSM conceptual framework and the research agenda. Third is a tool provided by the Epistemonikos Foundation, “Living Reviews”, allowing users to retrieve AI-generated evidence synthesis reviews from the PHSM bibliographic library. Lastly, the “PHSM Navigator” or “PHSM Recommendation Finder” provides a repository of PHSM-related recommendations in WHO guidelines (discussed below).

Here we analyze recent WHO recommendations on PHSM, making comparisons with pre-COVID-19 recommendations and thus exploring changes WHO has already made, and whether these are based on systematic, evidence-based evaluation of their overall effects on public health. Understanding how PHSM recommendations have changed and on what basis is important, since the development of recommendations that precede evidentiary evaluation may normalize interventions that could have negative public health impacts during future outbreaks.

## Methods

To capture recent WHO PPPR recommendations, documents were identified by scanning the titles of all publications on the WHO website (29) released between January 2017 and April 2025 for the terms non-pharmaceutical, measures, pandemic(s), epidemic(s), emergency, emergencies, and preparedness. Subsequently, to identify whether the documents provide PHSM recommendations for community settings, searches within the documents were conducted for keywords associated with PHSM (such as PHSM, measures, non-pharmaceutical, quarantine, school, masks, border, travel, distancing). Additionally, the “PHSM Recommendation Finder” of WHO’s “PHSM Knowledge Hub” was analyzed (30). The searches were last run on 25 May 2025.

PHSM were classified into the five categories following the taxonomy proposed in WHO’s conceptual framework: active case-finding and contact identification measures; personal protection measures; environmental measures; social measures, and; international travel and trade measures (23). PHSM not directly intended to physically restrict community (population) pathogen transmission were not considered in this analysis, including economic support, public information campaigns or building testing capacities, and rules limited to healthcare facilities. We excluded documents that dealt only with other domains of pandemic response such as clinical care, addressed policies in a limited geographical area or gave one-off ungeneralizable recommendations for an ongoing epidemic or pandemic, and briefing documents and progress and meeting reports. Disease-specific documents for influenza and COVID-19 were included if their recommendations remained active.

## Results

The search yielded 23 potentially relevant documents. Of these, 15 did not provide PHSM recommendations for community settings, although some are referred to in the discussion on overall guidance (25). A list of excluded documents can be found in the Supplementary material. The eight included documents, three from before 2020 (13, 31, 32) and five from after the WHO declared an end to the COVID-19 PHEIC (4, 5, 33–35), underwent content analysis to extract PHSM recommendations.

An additional search in the “PHSM Recommendation Finder” yielded 348 recommendations in total, all of them disease-specific. Over two-thirds of recommendations affect HIV, Tuberculosis and Malaria. While some argue that these should be labeled pandemics (36), they lack the property of rapid spread through and between populations characteristic of acute pandemic diseases (37). Furthermore, WHO’s PPPR agenda mainly aims at newly emerging infectious diseases (38). Of 25 recommendations addressing COVID-19, a majority concerned face masks. The most recent COVID-19 recommendations date from the “living guideline” on “Infection prevention and control in the context of COVID-19” from August 2023 (39), but a web search revealed a more up-to-date version from 21 December 2023, noting that “this update transitions the format from a living guideline to a guideline” (40). Although originally aimed at preventing nosocomial infections, IPC guidance on COVID-19 includes separate recommendations and good practice statements (GPS) for health-care settings and the community. It was therefore added to the eight publications identified in the keyword search.

Three of the resulting nine publications are explicitly labeled as guidelines and claim to follow the process in the WHO handbook for guideline development (16): the pre-COVID-19 2019 recommendations on pandemic influenza (13); the 2023 COVID-19 IPC guideline (40) and; the 2024 “WHO guideline on contact tracing” (35). The other five documents did not claim to follow a comparable systematic, evidence-based approach.

PHSM recommendations identified in the analyzed documents are listed in Table 1, grouped following WHO’s taxonomy of PHSM (23).

**Table 1:** PHSM recommendations in WHO documents.

Source	Active case-finding and contact identification measures	Personal protection measures	Environmental measures	Social measures	International travel and trade measures
Pandemic influenza risk management: a WHO guide to inform and harmonize national and international pandemic preparedness and response (2017)	Self-isolation for sick individuals is mentioned among the measures to be considered during an influenza pandemic. It is unclear whether “minimization of contact with others” only applies to sick individuals. It is further stated that the incubation period and the duration of infectiousness shall be used for planning the “length of isolation for cases (…) and the length of quarantine for contacts” (p. 48).	Not mentioned	Not mentioned	Measures to be considered during an influenza pandemic include “cancelation, restriction or modification of mass gatherings” (p. 40), and social distancing measures such as school closures and “adjusted working patterns” (p. 40). “Reduction of unnecessary travel and overcrowding of mass transport systems” (p. 36) may be considered during an influenza pandemic, but it is not clear whether these shall be enforceable.	Not mentioned
Managing epidemics: Key facts about major deadly diseases (2018)	Active case finding / contact tracing recommended for several diseases (Ebola, Lassa fever, Crimean-Congo haemorrhagic fever, MERS, Cholera, Mpox). Isolation of patients is recommended for several diseases including seasonal influenza. Quarantine is described as “unacceptable to many populations today” (p. 26).	Appropriate PPE is mentioned in the context of healthcare for several diseases, including for seasonal influenza patients. Wearing facemasks when sick considered as an “extreme measure” during severe influenza pandemics (p. 146). Several personal protection tools are recommended against vector-borne diseases (bednet, repulsive, window screen, insecticide sprays, electric devices). Masks, gloves and gowns are further recommended when slaughtering and butchering animals.	Different environmental measures are recommended for vector-borne diseases (e.g., insecticides, eelimination of tick, mosquito and flea breeding sites, mechanical trapping)	“Social distancing” is considered for seasonal and pandemic influenza. In the section on seasonal influenza, social distancing is said to include “isolation of patients, staying at home when sick, and school closure” (p. 136). In the section on pandemic influenza people who fall sick are also advised more broadly to distance themselves from others. During severe influenza pandemics, school closures and decreasing the amount of contacts among people are considered as possible but “extreme” measures.	Border control measures such as entry or exit screening or border closures are explicitly not recommended for seasonal influenza and not mentioned for other diseases.
Non-pharmaceutical public health measures for mitigating the risk and impact of epidemic and pandemic influenza (2019)	“Voluntary isolation at home of sick individuals” (p. 42) is recommended during all influenza epidemics and pandemics. Contact tracing and quarantine of exposed individuals are not recommended in any circumstances. However, active contact tracing “could be considered in some locations and circumstances to collect information on the characteristics of the disease and to identify cases, or to delay widespread transmission in the very early stages of a pandemic in isolated communities” (p. 38).	A disposable surgical mask is recommended to be worn at all times by symptomatic individuals when in contact with others. Wearing of masks by asymptomatic people is conditionally recommended in severe epidemics or pandemics Hand hygiene and respiratory etiquette are further recommended as personal protection measures during epidemics or pandemics of any severity.	Surface and object cleaning and increased ventilation are recommended during pandemics and epidemics of any severity. This is despite surface and object cleaning also being described as “ineffective in reducing respiratory disease transmission in the community” (p. 30), and increased ventilation as lacking evidence to reduce transmission, but it is described as a measure with no major disadvantages. UV light and modifying humidity are not recommended in any circumstances.	School and workplace measures and measures to avoid crowding (e.g., ban of mass gatherings) are conditionally recommended. School measures can include, e.g., “stricter exclusion policies for ill children, increasing desk spacing, reducing mixing between classes, and staggering recesses and lunchbreaks” (p. 52). Workplace measures can include “encouraging teleworking from home, staggering shifts, and loosening policies for sick leave and paid leave” (p. 56). “Coordinated proactive school closures or class dismissals” (p. 52) are suggested during severe epidemics or pandemics. In extraordinarily severe pandemics, “extreme measures such as workplace closures can be considered” (p. 56). “Internal travel restrictions are conditionally recommended during an early stage of a localized and extraordinarily severe pandemic for a limited period of time” (p. 65).	Entry and exit screening for infection in travelers is not recommended. Border closure is generally not recommended “unless required by national law in extraordinary circumstances during a severe pandemic” (p. 68). They “may be considered only by small island nations in severe pandemics and epidemics, but must be weighed against potentially serious economic consequences” (p. 4).
Managing epidemics: key facts about major deadly diseases, 2nd edition (2023)	Active case finding / contact tracing recommended for several diseases (Ebola, Lassa fever, Crimean-Congo haemorrhagic fever, MERS, Cholera, Mpox). Isolation of patients is recommended for several diseases including seasonal influenza. Quarantine of contacts is recommended for MERS, COVID-19 “Implement restrictions on freedom of movement only if less restrictive measures are unlikely to be as effective in achieving important public health objectives, and with the fewest constraints reasonably possible, ensuring humane conditions and equitable application” (p. 45).	Mask-wearing is mentioned as a personal protection measure without specifications for influenza, COVID-19, mpox. PPE without further specification is mentioned for animal influenza. For seasonal influenza, it is further specified that well-fitted masks should be worn by symptomatic individuals when in contact with others. For COVID-19, universal masking is recommended in health care settings in areas of widespread community transmission. Use of masks is further adviced to health care workers treating Lassa Fever, CCHF, MERS, mpox.	Cleaning and effective ventilation is recommended against seasonal and pandemic influenza, COVID-19, mpox. Different environmental measures are recommended for vector-borne diseases (e.g., insecticides, elimination of tick, mosquito, flea, and sand flies breeding sites, mechanical trapping)	Workplace measures and closure and avoiding overcrowding (e.g., bans of mass gatherings) are included in the general PHSM box. For influenza, both seasonal and pandemic, school and workplace measures and closures, and avoiding overcrowding are conditionally recommended depending on the severity of the epidemic or pandemic. Furthermore, maintaining a distance in public or workplaces is recommended. For COVID-19, this is to be at least 1 meter. Furthermore, possible physical distancing measures against COVID-19 include “regulating the number and flow of people attending gatherings, maintaining distance in public or workplaces, school closure or class suspensions, encouraging remote working options and online education” (p. 180). Internal travel restrictions are listed in a box of PHSM without further recommendations on when and whether to apply them. It is advised to “implement restrictions on freedom of movement only if less restrictive measures are unlikely to be as effective in achieving important public health objectives, and with the fewest constraints reasonably possible, ensuring humane conditions and equitable application” (p. 45).	Border control measures such as entry or exit screening or border closures are explicitly not recommended for seasonal influenza and not mentioned for other diseases.
Infection prevention and control in the context of COVID-19: a guideline (2023)	Isolation is recommended for healthcare workers, but not explicitly for others, although a good practice statement on mask wearing mentions an “isolation period” (p. 19). Quarantine is mentioned only under certain conditions for health care workers.	Masks are strongly recommended in the community for everyone 12 or older “when in crowded, enclosed, or poorly ventilated spaces; following recent exposure to COVID-19 (according to the WHO definition) when sharing a space with others; when sharing a space with a person who displays signs or symptoms of COVID-19 or is COVID-19-positive; for individuals at high risk of severe complications from COVID-19”. In situations not covered by the strong recommendation, a “risk-based approach” to masking shall be followed, informed by different factors including epidemiological trends. Children aged 6 to 11 are recommended to wear masks in areas where there is “known or suspected community transmission”, “indoor settings where ventilation is known to be poor or cannot be assessed, or the ventilation system is not properly maintained, regardless of whether physical distancing of at least 1 meter can be maintained”, or even in all other indooe settings when distance cannot be maintained. WHO further recommends against wearing masks for children 5 or younger, or for anyone during vigorous-intensity physical activity. Good practice statements recommend individuals with symptoms or who tested positive should wear a medical mask when sharing a space with others. Other good practice statements recommend against wearing a mask for children with certain health conditions that make doing so difficult, but leave the wearing of masks by children at risk of severe COVID-19 at the discretion of their medical provider.	A Good Practice Statement advises households and community settings to “follow routine environmental cleaning and disinfection practices” (p. 20).	Social measures are only listed as examples in the definition of PHSM.	International travel measures are only listed as examples in the definition of PHSM.
WHO benchmarks for strengthening health emergency capacities (2023)	Surveillance and contact tracing are mentioned as examples of PHSM that are expected to “play an immediate and critical role throughout the different stages of health emergencies” (p. 286).	Mask wearing is mentioned as example of PHSM that are expected to “play an immediate and critical role throughout the different stages of health emergencies” (p. 286).	Not mentioned	“Social measures, such as restricting mass gatherings and modifying school and business openings and closures” are mentioned as examples of PHSM that are expected to “play an immediate and critical role throughout the different stages of health emergencies” (p. 286). “Policies for alternative modalities to deliver school meals and other school-linked and school-based social protection when schools are closed due to emergencies” (p. 325).	States are to “develop or update legislation (relevant to screening, quarantine, testing, contact tracing, etc.) to enable the implementation of international travel related measures” (p. 270). To meet the “demonstrated capacity” benchmark, States must “establish isolation units to isolate and quarantine suspected human or animal cases of communicable diseases” (p. 267) and are further expected to perform simulation exercises including “on different components of international travel related measures (such as entry/exit screening, contact tracing, quarantine)” (p. 271).
Learnings from COVID-19 for future respiratory pathogen pandemic preparedness: a summary of the literature (2024)	Quarantine is not explicitly recommended, but countries “should ensure that pandemic plans explicitly account for the unique challenges faced by vulnerable populations when (…) complying with (…) isolation and quarantine measures” (p. ix). Furthermore, a box listing examples of successful leveraging of existing health programs includes the case of South Africa where healthcare workers performed house-to-house searches for COVID-19 cases (p. 18).	No measures are explicitly recommended, but masking is listed as an example of PHSM (p. 22).	“Improving indoor environmental quality in residential, school and childcare, workplace and community gathering settings can mitigate the transmission of future respiratory pathogens with pandemic potential” (p. 21).	No measures are explicitly recommended, but countries “should ensure that pandemic plans explicitly account for the unique challenges faced by vulnerable populations when navigating travel restrictions; complying with lockdown, isolation and quarantine measures” (p. ix).	No measures are explicitly recommended, but countries “should ensure that pandemic plans explicitly account for the unique challenges faced by vulnerable populations when navigating travel restrictions” (p. ix).
Preparedness and resilience for emerging threats: Module 1: planning for respiratory pathogen pandemics. (2024)	Isolation, contact tracing and quarantining of exposed individuals “will likely be needed to cut transmission chains” and containing an outbreak. These measures are also included in PHSM that can “reduce transmission and spread of respiratory pathogens and minimize public health impact” (p. 23).	Mask-wearing, hand hygiene, and respiratory etiquette are briefly mentioned as a measure that can reduce transmissions and spread of respiratory pathogens and minimize public health impact.	Cleaning, disinfection and indoor ventilation are briefly mentioned as a measure that can reduce transmissions and spread of respiratory pathogens and minimize public health impact. Indoor air quality interventions are singled out as particularly important, calling for “a new era of pandemic-resilient buildings, environmental sustainability with proactive disease control, and rational use of indoor ventilation, filtration and other scalable interventions” (p. 24).	Physical distancing measures that “can reduce transmission and spread of respiratory pathogens and minimize public health impact” (p. 23) include regulating the number and flow of people attending gatherings, maintaining distance in public places, schools or workplaces. Domestic movement restrictions are mentioned as one of several PHSM that that “can reduce transmission and spread of respiratory pathogens and minimize public health impact” (p. 23).	Recommends to “build on plans and procedures established during COVID-19” including “surveillance and case management at points of entry and onboard conveyances” (p. 43). “Exit/entry screening for signs and symptoms, targeted testing and quarantine of travelers” should be included in contingency plans.
WHO guideline on contact tracing (2024)	Intensified contact person identification and active follow-up of contacts is recommended in populations at risk of infectious diseases. Isolation and quarantine are not part of the recommendations but named as potential measures.	Not mentioned	Not mentioned	Not mentioned	Not mentioned

## Discussion

In the following, we discuss our observations grouped around four areas: first, we show an evident normalization of PHSM formerly advised against, most notably quarantine, before discussing the normalization of universal masking separately. A third section will explore issues around uncertainty, the precautionary principle and mitigation of harm from PHSM. Lastly, we will address the newly emerged topic of infodemic management.

### Normalization of quarantine and other PHSM irrespective of evidence

A normalization of PHSM applied during the COVID-19 pandemic is evident throughout recent WHO publications. This is well exampled by comparing the updated “Managing Epidemics” handbook (4) with its previous edition from 2018 (32). Though targeted mainly at WHO country office staff advising ministries of health, the document’s non-technical language and easy navigation make it accessible to a wider audience. A new section on PHSM calls for “*tailored and evidence-informed combinations of different measures*”. Quarantine and movement restrictions are portrayed in a much less critical light than previously. While the 2018 version stated:

“…many traditional containment measures are no longer efficient. They should therefore be re-examined in the light of people’s expectations of more freedom, including freedom of movement. Measures such as quarantine, for example, once regarded as a matter of fact, would be unacceptable to many populations today” (32).

The revised version changes this to:

“… many traditional containment measures are challenging to put in place and sustain. Measures such as quarantine can be at odds with people’s expectations of more freedom, including freedom of movement. Digital technologies for contact tracing became common in response to COVID-19. These, however, come with privacy, security and ethical concerns. Containment measures should be re-examined in partnership with the communities they impact” (4).

Containment is “challenging” rather than “no longer efficient”, while quarantine is no longer “unacceptable” (4).

PHSM have also now also entered the “WHO benchmarks for strengthening health emergency capacities” (5). The benchmarks were first issued in 2019 as a tool for States to monitor their progress toward fulfilling core capacities of the International Health Regulations (IHR) (41). A new benchmark reads “Leadership and governance dedicated to public health and social measures (PHSM) is in place in relevant sectors, at all levels and between levels”. The new document considers PHSM to “range from surveillance, contact tracing, mask wearing and physical distancing to social measures, such as restricting mass gatherings and modifying school and business openings and closures”, and to “play an immediate and critical role”. States are expected to “review and adjust PHSM policies and implementation based on timely and regular assessment of data”, to “routinely monitor PHSM”, and to “establish whole-of-government mechanisms” to implement them (5). Benchmarks of control on points of entry have been expanded substantially, introducing isolation, screening, contact tracing and quarantine for which States are expected to “develop or update legislation” to enhance control of international travel. To meet the benchmark, States must establish isolation and quarantine units for human and animal communicable diseases, and perform simulation exercises to demonstrate they are functional (5).

Consistent with the managing epidemics handbook, WHO’s Preparedness and Resilience for Emerging Threats (PRET) initiative provides PPPR guidance grouping pathogens based on their ways of transmission. PRET’s first module, for respiratory pathogen pandemics, claims that contact tracing and quarantining of exposed individuals “will likely be needed to cut transmission chains” and can reduce transmission and minimize public health impact. Parallel to the provisions in the updated benchmarks, PRET also notes that contingency plans should include “exit/entry screening for signs and symptoms, targeted testing and quarantine of travelers” (34).

The new WHO guideline on contact tracing recommends “intensified contact person identification”, defined as “in-depth investigations of cases conducted by a public health professional, usually at point of diagnosis or care” (35), involving active follow-up with contact persons. Lastly, the guideline recommends testing to be added to contact tracing, distinguishing between “test to trace” and “test to release” functions. The latter, defined as “testing to clear contact persons or have a follow-up period end sooner” indicates the possibility of quarantine although the guidelines do not include any such specific recommendations.

Table 2 summarizes changes in PHSM recommendations for respiratory pathogens that directly contrast earlier editions of the same document or the recommendations WHO gave in 2019 for responding to pandemic influenza, where contact tracing, quarantine of exposed individuals, and border screening were not recommended “in any circumstances”, with even isolation of symptomatic individuals recommended to be voluntary (13). To allow for comparability, Table 2 only includes recommendations that do not exclusively apply to COVID-19.

**Table 2:** Changes in PHSM recommendations for respiratory pathogens.

Source	New recommendation	Change to pre-COVID recommendations
Managing epidemics: key facts about major deadly diseases, 2nd edition (2023)	Wearing a mask is listed as a personal protection measure in community settings for several diseases, including for influenza. Regarding seasonal influenza, the handbook suggests that “[w]ell-fitted masks should be worn by symptomatic individuals when in contact with other individuals” (p. 146).	In the first edition of the same handbook, wearing facemasks when sick was considered as an “extreme measure” during severe influenza pandemics (p. 146). A recommendation for influenza patients to wear a mask was restricted to healthcare settings (p. 136).
WHO benchmarks for strengthening health emergency capacities (2023)	PHSM ranging from “surveillance, contact tracing, mask wearing and physical distancing to social measures, such as restricting mass gatherings and modifying school and business openings and closures” are said to “play an immediate and critical role throughout the different stages of health emergencies” (p. 286). States are expected to develop legislation to enable the implementation of international travel related measures, (i.e., “screening, quarantine, testing, contact tracing, etc.”) (p. 270) and to “establish isolation units to isolate and quarantine suspected human or animal cases of communicable diseases” (p. 267).	PHSM were not mentioned in the first version of the benchmarks. Contact tracing, quarantine of exposed individuals, and entry and exit screening were all “not recommended in any circumstances” in 2019 guidance for influenza pandemics (p. 3).
Preparedness and resilience for emerging threats: Module 1: planning for respiratory pathogen pandemics. (2024)	“A suite of measures will likely be needed to cut transmission chains including extensive testing, case isolation, contact tracing and quarantining of exposed individuals” (p. 23). International border measures such as “Exit/entry screening for signs and symptoms, targeted testing and quarantine of travelers” should be included in contingency plans for respiratory pandemics (p. 42).	Contact tracing, quarantine of exposed individuals, and entry and exit screening were all “not recommended in any circumstances” in 2019 guidance for influenza pandemics (p. 3).
WHO guideline on contact tracing (2024)	Intensified contact person identification and active follow-up of contacts is recommended in populations at risk of infectious diseases.	Contact tracing was “not recommended in any circumstances” in 2019 guidance for influenza pandemics (p. 3).

The first three documents featured in Table 2 do not use any references to substantiate their recommendations. The only recent PHSM document to follow the WHO Handbook for Guideline Development (16) is the mentioned guidance on contact tracing. However, the evidence for its recommendations is rated as being of “very low certainty” according to the GRADE approach, meaning that further research is very likely to change them (35).

Nonetheless, WHO claims in the executive summary to its report on the role of social protection in reducing the burden of PHSM during the COVID-19 pandemic that “PHSM were effective in curbing the outbreak” (42). While an in-depth review of restrictive PHSM effectiveness would go beyond the scope of this article, it is worthwhile to discuss the references used by WHO to claim that PHSM “were effective in significantly reducing the transmission of SARS-CoV-2” (43–45) as well as deaths due to COVID-19 (46).

The first reference is a WHO-supported systematic review of systematic reviews by Fadlallah et al. on the effects of PHSM during COVID-19 (43). It included 94 reviews synthesizing over 1,000 primary studies and found predominantly no or very low-certainty evidence regarding both intended and unintended effects. Low certainty evidence was found in favor of routine testing of residents and staff in long-term care settings. Symptom- or exposure-based screening of travelers at borders was said to have reduced imported cases with moderate certainty, but based solely on one modeling study from China. Screening for symptoms among air travelers and quarantining travelers was said to shift pandemic development positively with low certainty, despite unknown transmission impact. The review highlights the difficulty of attributing effectiveness to specific measures, leading Fadlallah et al. to the overall conclusion that there is “low-certainty evidence that multicomponent interventions may reduce the transmission of COVID-19 in different settings” (43). This tempers WHO’s claim that measures considered “unacceptable” until recently (32) were “effective in curbing the outbreak” (42). The second reference is a study combining a synthesis of systematic reviews with a Delphi technique, i.e., expert survey (45). Notably, the interviewed experts were selected as those who have been actively involved in COVID-19 response policies. Thus, officials from national Ministries of health and public health institutes were evaluating their own policies.

The third reference is the executive summary of a compendium of evidence reviews conducted by the Royal Society, concluding that stringent lockdowns were effective in reducing SARS-CoV-2 transmission (44, 47–53). However, 334 of 338 included observational studies were ranked as being of low or very low quality, including many modeling studies resting on unproven assumptions. Despite the low certainty of evidence, the Royal Society review found quarantining international travelers to be effective in some contexts, namely at the beginning of the outbreak, or when applied rigorously alongside domestic measures to keep SARS-CoV-2 transmission at very low levels (51). Another review suggests that testing, tracing and isolation successfully reduced transmission in some contexts (50).

In addition to the six systematic reviews on different classes of PHSM, the Royal Society report highlighted three locations that successfully contained SARS-CoV-2 transmission at very low levels for approximately 18 months: Hong Kong, New Zealand, and South Korea. However, of the three examples, only New Zealand sustained an exceptionally low mortality, while in Hong Kong and South Korea, infections and deaths peaked shortly after, leading to total mortality figures comparable to those of other high-income countries (54). While the featured case studies illustrate that suppressing a virus like SARS-CoV-2 is possible under the right circumstances, it would be equally possible to highlight the Nordic countries, which had some of the lowest cumulative excess mortality rates over the entire pandemic globally despite some of the least restrictive interventions (55).

The study WHO cites to claim the effectiveness of PHSM to reduce COVID-19 deaths is equally restricted to the first pandemic wave (46). It does not find a significant effect of the major restrictive measures now endorsed by WHO (border screening, quarantine), but only of earlier school and workplace closures, which themselves have major and unmeasured long term educational and economic ramifications.

In summary, the evidence on which WHO bases their updated recommendations is dominated by low-quality studies with often contradictory results. While some studies of PHSM suggest their short-term effectiveness in lowering transmission, what has worked in a high-income island nation like New Zealand cannot be emulated in many other contexts. A rigorous evaluation would also need to account for the magnitude of any effect. For example, a meta-analysis by Herby et al. estimates that the average lockdown in Europe and North America reduced COVID-19 mortality in the spring of 2020 by just 3 % (56).

A narrow focus on SARS-CoV-2 transmission and mortality is also problematic as some measures can cause significant collateral damage, e.g., by reducing access to medical care, impairing mental health or impacting other social determinants of health (9, 11, 57, 58). Overall, the evidence cannot provide the medium and longer term efficacy and adverse outcome metrics necessary for evaluating policies with complex health, economic, and other societal consequences (54, 59).

### Normalization of universal masking

Universal masking is the only post-pandemic recommendation identified which builds on evidence rated as being of low-to-moderate certainty. WHO’s COVID-19 IPC Guidelines (40) thus state that “core PHSM (for example, mask use, physical distancing) should be maintained in priority groups, settings and situations, even during periods of low transmission”. Given that SARS-CoV-2 is now endemic, a literal adherence to the IPC guidelines would require everyone aged 6 or older to wear a mask in all indoor spaces where a distance of 1 meter to others cannot be upheld at all times. People aged 60 or older, or those with underlying comorbidities, are recommended to wear a mask irrespective of their environment. While the 2018 “Managing Epidemics” handbook still referred to masking of sick people as an “extreme measure” to be considered in severe pandemics (32), the 2023 update recommends wearing masks for everyone, irrespective of health status, even against seasonal influenza, and normalizes masking by listing it together with hand hygiene (4).

However, the effectiveness of masking policies remains disputed. A Cochrane review of randomized controlled trials (RCTs) found no significant effect of community mask use on respiratory illness (60), although the largest trial, a cluster RCT in Bangladesh by Abaluck et al., recorded a reduction in illness (61, 62), though this study has also been criticized (63–65). Two other RCTs found no significant protective effect on mask wearers (66, 67), although this does not provide evidence regarding “source control” (i.e., masking infected individuals to prevent spread to others). Tightness of fit, frequency of replacement, and other aspects of compliance are difficult to measure, but also highly relevant to real-life effectiveness and likely to vary widely in place and time (68). The WHO-supported PHSM review by Fadlallah et al. notes that universal masking may reduce the risk of COVID-19 outcomes based only on critically low-confidence reviews from 2020 (43). The Royal Society review on masks similarly concluded that masks and mask mandates reduced SARS-CoV-2 transmission based almost exclusively on observational studies at critical risk of bias (48). If universal masking had any effect on SARS-CoV-2 transmission, it was relatively small, as it is not clearly visible in international comparisons. Sweden, as one of few countries to never have a mask mandate, had one of the lowest excess mortality rates over the course of the pandemic (55).

Any policy on widescale masking also needs to take into account potential physical, psychological and social harms, and ethical implications (69, 70). The COVID-19 IPC guideline indicates that WHO rates the harms of masks to be very small, and only directly suggests against wearing masks during vigorous-intensity physical activity. WHO found no evidence for serious harms in adults in community settings “although bothersome harms were common”. Indeed, large numbers of people reported difficulties in breathing in some studies (71), while others suggest that masking may reduce cognitive performance (72). Even if masking policies allow for exemptions in individuals with difficulties wearing a mask, universal masking can have effects on others” (76) wellbeing (e.g., to those hard of hearing) (73). Of particular concern are potential detrimental effects on the wellbeing and development of children (69, 74). Environmentally, they add substantially to global plastic pollution (75).

### Uncertainty, precaution and mitigation

Several documents express WHO’s awareness of the adverse effects of PHSM, although the focus within these documents remains on mitigation of potential outbreak risks rather than prevention of secondary harms caused by PHSM. For example, the report on learnings from COVID-19 that WHO commissioned from the Johns Hopkins Center for Health Security notes that “the implementation of PHSM imposed a socioeconomic burden on people, and this burden often led to unintended consequences for health and health equity by adversely impacting the social determinants of health” (33). Moreover, the updated benchmarks list harms including increasing loneliness, food insecurity, the risk of domestic violence, and reducing household income and productivity (5). WHO’s PHSM Monitoring guidance recommends countries “strike a balance between public health and economic well-being” (25), and the recent contact tracing guideline notes that it is “crucial to take a holistic view”, weighing benefits against health, social and economic costs for individuals and society (25). To this end, WHO proposes integrating epidemiological and economic modeling (76), and has published an evidence review on the role of social protection in reducing the PHSM burden (42).

In its Benchmarks for strengthening emergency capacities (5), a new benchmark reads “The protection of livelihoods, business continuity and continuity of education and learning systems is in place and functional during health emergencies”. Here, disruptions, particularly to schooling, seem to be expected, and the ability to address the requirements of this benchmark will clearly be unequal between countries, driving overall inequality. WHO’s review of learnings from COVID-19 identified the need for a strong social safety net. A specification that “Countries (…) should ensure that pandemic plans explicitly account for the unique challenges faced by vulnerable populations when navigating travel restrictions; complying with lockdown, isolation and quarantine measures; and accessing health and social services” recognizes the global crisis of livelihoods, business continuity and continuity of education caused by the COVID-19 response (33). However, the proposed “[r]obust social safety net programs” are unlikely to fully offset non-material harms (33, 77). Large scale social safety programs are also typically not feasible in low-income settings. Lastly, the associated fiscal expansion contributed to increases in inflation and further impoverishment (78), and mitigation will have to somehow be implemented in the presence of impaired economies and restricted government services. Thus, WHO’s mitigation recommendations seem poorly tied to reality.

An awareness of the adverse effects of PHSM highlights that pandemic response will necessitate rapid decision making under conditions of uncertainty requiring trade-offs while producing subsequent secondary harms. During the COVID-19 response WHO had the difficult task of both providing immediate pandemic guidance to mitigate unknown risks associated with SARS-CoV-2 while also not causing greater social and economic harms. However, like many governments at the time, WHO often invoked two principles to justify PHSM despite anticipated secondary harms, the “precautionary principle” and the “principle of no regrets”. The former principle has often been quoted to justify the implementation of unprecedented precautionary measures to protect public health from immediate but unknown risks under conditions of uncertainty, allowing for “states of exception” in order to limit transmission and mitigate direct outbreak harms (79). For example, the discussed PRET module states that “a precautionary approach to infection prevention early in the event will save lives”, advising decision makers to “[b]e ready to apply stringent PHSM, but for a limited time period in order to minimize associated unintended health, livelihood and other socio-economic consequences” (34). The “no regrets” principle is a related concept, which suggests that it is appropriate to overprepare during a crisis rather than wait for additional evidence-based considerations. As the name suggests, one should not regret decisions made in good faith even if those decisions prove to be wrong (80–82).

Although a level of inaccuracy in decision making is understandable when faced with risks and uncertainty, in the context of new PHSM guidelines, two reflections are worth noting. First, traditional understandings of the precautionary principle are negative, not positive (83). This means that problems and risks are to be avoided by not engaging in specific activities until it is certain that those activities will not lead to potentially foreseeable harm (79). In the case of PHSM, a more traditional understanding of the precautionary principle could arguably suggest that it is thus necessary to err on the side of caution negatively by refraining from PHSM actions that will have foreseeable secondary health and social harms. Second, although the principle of no regrets does absolve decision makers from ethical culpability when decisions were made in good faith, it does not absolve them from recognizing the unintended harms associated with their decisions after the fact nor to ignore important lessons from those outcomes. In other words, the principle does not suggest that one should never recognize regrets (what one should have done otherwise) after access to better information.

Consequently, a key lesson from COVID-19 is that it requires the weighting of other known or highly likely harms associated with PHSM measures, such as lost education, denied access to routine healthcare, increased wealth gaps, social isolation and mental illness, increased poverty, increased sovereign debt accumulation, and general GDP decline. Whereas early precautionary measures may have been justified in the face of uncertainty and perceived SARS-CoV-2 risk, over time, precaution and the mitigation of harm required better adaptation as information improved (84). Unfortunately, in the case of emerging PHSM guidance and COVID-19 learnings, these concerns have received recognition while remaining largely undervalued.

### PHSM, infodemic management and public trust

In its COVID-19 learnings report, WHO further states that “Pandemic plans should also explicitly account for the threats posed by misinformation and disinformation about (…) government decisions regarding pandemic mitigation and response” (33). WHO encourages States to set up a team for the management of “infodemics”, defined as “an overabundance of information, accurate or not, in the digital and physical space, accompanying an acute health event such as an outbreak or epidemic”. A new segment on “infodemic in practice” in the updated “Managing Epidemics” publication highlights listening to concerns, communicating risk, and using “evidence and facts” to “debunk misinformation and disinformation that could have a negative health impact on people and communities, while respecting their freedom of expression” (4).

The term “misinformation” was frequently used during the COVID-19 pandemic to dismiss or even censor valid scientific perspectives (11, 85). This risks neglecting the heterogeneity of scientific viewpoints and depoliticising policy (86–89). As demonstrated during COVID-19 when policy was often based on epidemiological models resting on unproven assumptions (11, 90), a policy of excluding contrary opinion carries high risk. A 2024 WHO publication advocating for strengthening the role of integrated epidemiological and economic modeling for pandemic response carries this risk forward (76).

A perception of exclusion of alternate opinion also risks public trust (87, 91). The real risk of COVID-19 for many demographics was exaggerated manyfold in public perceptions, partly because people used the unfamiliar stringency of PHSM as a heuristic to estimate the risk posed by the disease, and partly due to deliberate exaggeration by public health authorities (92, 93). The Scientific Advisory Group for Emergencies (SAGE) in the UK advised their government that “the perceived level of personal threat needs to be increased among those who are complacent, using hard-hitting emotional messaging” (94). When combined with a perception of suppression of alternate, more moderate opinion, a resultant loss of trust is likely to be counterproductive. There also remains a major evidence gap on whether such incitement of fear and stress results in an overall public health good.

## Conclusion

Our research highlights the adoption and normalization of several COVID-19 PHSM within post-COVID-19 WHO PPPR recommendations, despite poor quality evidence on costs and benefits. As chronicled during COVID-19, PHSM are not benign interventions, with potentially harmful social, economic, psychological and health effects. A proper evaluation of the evidence to support PHSM is therefore essential to guide future policy. It would be prudent to thoroughly understand their impact during the COVID-19 pandemic and emerging longer-term impacts.

The one-size-fits-all approach suggested by these policy changes, and evidenced in the COVID-19 response, is a major break from more orthodox and targeted approaches that consider local context in weighing risks together with benefits. WHO recommendations are therefore likely to exacerbate inequalities, including recommendations on mitigating PHSM harm. Calm rigor, rather than a rush to publication, would provide a path to better PPPR and public health outcomes.

## Data Availability

The original contributions presented in the study are included in the article/Supplementary material, further inquiries can be directed to the corresponding author.
