# Supplementary material for: WHO’s pandemic response recommendations after COVID-19: lessons learned or learnings lost?
Source: Front Public Health. 2025 Oct 28;13:1664330. doi: 10.3389/fpubh.2025.1664330 (PMC12602469; doi:10.3389/fpubh.2025.1664330)
Supplement: Supplementary file 1 [file Table_1.docx]

Excluded documents:

| no PHSM | 2017 | https://iris.who.int/bitstream/handle/10665/254883/9789241511827-eng.pdf?sequence=1 | A strategic framework for emergency preparedness |
| --- | --- | --- | --- |
| no PHSM | 2018 | https://www.who.int/publications/i/item/WHO-WHE-IHM-GIP-2018.1 | Essential steps for developing or updating a national pandemic influenza preparedness plan |
| no PHSM | 2020 | https://www.who.int/publications/i/item/9789240006232 | Multisectoral preparedness coordination framework |
| PHSM only in health care | 2021 | https://www.who.int/publications/i/item/9789240032729 | Framework and toolkit for infection prevention and control in outbreak preparedness, readiness and response at the national level |
| no PHSM | 2021 | https://www.who.int/publications/i/item/9789240037830 | Framework for strengthening health emergency preparedness in cities and urban settings |
| no PHSM | 2021 | https://www.who.int/publications/i/item/9789240037182 | WHO guidance on preparing for national response to health emergencies and disasters |
| PHSM only briefly mentioned | 2021 | https://www.who.int/publications/i/item/9789240015081 | Everyone’s business: Whole-of-society action to manage health risks and reduce socio-economic impacts of emergencies and disasters: Operational guidance |
| scenarios, not recommendations | 2022 | https://www.who.int/publications/i/item/9789240052093 | Imagining the future of pandemics and epidemics: a 2022 perspective |
| no detailed PHSM recommendations | 2023 | https://www.who.int/publications/i/item/9789240063587 | Early warning alert and response (EWAR) in emergencies: an operational guide |
| part of PRET, no detailed recommendations | 2023 | https://www.who.int/publications/i/item/9789240084513 | A checklist for respiratory pathogen pandemic preparedness planning |
| no PHSM | 2024 | https://www.who.int/publications/i/item/9789240058064 | Emergency response framework (‎ERF)‎, Edition 2.1 |
| No detailed PHSM recommendations | 2024 | https://www.who.int/publications/i/item/9789240090880 | Strengthening pandemic preparedness and response through integrated modelling |
| No detailed PHSM (implementation) recommendations | 2024 | www.who.int/publications/i/item/9789240094444 | Global guidance on monitoring public health and social measures policies during health emergencies |
| No detailed PHSM recommendations | 2024 | https://www.who.int/publications/i/item/9789240096134 | Defining community protection: a core concept for strengthening the global architecture for health emergency preparedness, response and resilience |
| No detailed PHSM recommendations | 2025 | https://www.who.int/publications/i/item/9789240094246 | A framework for tracking global progress on preparedness for respiratory pandemics |
